# Avenanthramides: Unique Bioactive Substances of Oat Grain in the Context of Cultivar, Cropping System, Weather Conditions and Other Grain Parameters

**DOI:** 10.3390/plants10112485

**Published:** 2021-11-17

**Authors:** Václav Dvořáček, Michal Jágr, Anna Kotrbová Kozak, Ivana Capouchová, Petr Konvalina, Oldřich Faměra, Petra Hlásná Čepková

**Affiliations:** 1 Crop Research Institute, Drnovská 507, 161 06 Prague, Czech Republic; dvoracek@vurv.cz (V.D.); jagr@vurv.cz (M.J.); anna.kotrbova@vurv.cz (A.K.K.); 2 Department of Agroecology and Crop Production, Faculty of Agrobiology, Food and Natural Resources, Czech University of Life Sciences Prague, Kamýcká 129, 165 00 Prague, Czech Republic; capouchova@af.czu.cz; 3 Department of Agroecosystems, Faculty of Agriculture, University of South Bohemia in České Budějovice, Studentská 1668, 370 05 České Budějovice, Czech Republic; konvalina@zf.jcu.cz; 4 Department of Food Science, Faculty of Agrobiology, Food and Natural Resources, Czech University of Life Sciences Prague, Kamýcká 129, 165 00 Prague, Czech Republic; famera@af.czu.cz

**Keywords:** avenanthramides, oat, cultivars, grain quality, cropping system, weather conditions

## Abstract

Our study was focused on the evaluation of the content of a wider spectrum of eight avenanthramides (AVNs) as unique components of oat grain under the effects of four selected factors (cultivar, locality, cropping system, and year). The weather effects on changes in the AVN content and their relationship to other important parameters of oat grain were further evaluated in more detail. A sensitive UHPLC system coupled with a QExactive Orbitrap mass spectrometer was used for AVN quantification. AVNs confirmed a high variability (RDS = 72.7–113.5%), which was dominantly influenced by the locality and year factors. While most AVN types confirmed mutually high correlations (r = 0.7–0.9), their correlations with the other 10 grain parameters were lower (r ≤ 0.48). Their significant correlations (0.27–0.46) with β-D-glucan could be used in perspective in breeding programs for the synergetic increase of both parameters. PCA analysis and Spearman correlations based on individual cultivars confirmed a significant effect of June and July precipitation on the increase of Σ AVNs. However, the results also indicated that higher precipitation can generate favorable conditions for related factors, such as preharvest sprouting evoking a direct increase of AVNs synthesis in oat grain.

## 1. Introduction

Oat (*Avena sativa* L.), with a current production share of about 2.3% of all cereals in the European Union, can be considered as a minor cereal compared to wheat or barley production [1]. The genus Avena includes about 70 species. The most important cultivated oat belongs to the hexaploid species *Avena sativa* L. (2n = 6x = 42; AACCDD). Within this species, two different types of cultivars were cultivated: oat with hulled and oat with naked grain [2]. The commercial oat cultivars with naked grain are referred to in some studies [3,4] as a separate species of *Avena nuda* L. Nevertheless, the species *A. nuda* belongs taxonomically to diploid oats which are not commonly cultivated [2].

The nutritionally high-value composition of oat grain has found its application in both livestock fattening and human nutrition. Oat consumption in the human diet has been increased because of health benefits associated with advantageous composition in macronutrients: (i) lipids with a high degree of unsaturation, including oleic and linoleic acids (about 40% and 36% of total fatty acids, respectively), (ii) proteins with a favorable composition of essential amino acids, and (iii) dietary fibers with a high content of β-glucan (2–8.5% w/w of oat seed) [3]. Since 2009 (Commission Regulation No. 41/2009) and 2013 (Food and Drug Administration FDA), oat products can be sold as gluten-free in several countries provided a gluten contamination level below 20 ppm [5].

Oat grain also contains a broad range of phenolic compounds which are mainly concentrated in the outer layer of the kernel. These compounds are secondary products of plant metabolism. They have high levels of antioxidant activity and several studies have confirmed their beneficial effect on cardiovascular disease (CVD), diabetes, inflammatory bowel disease (IBD), obesity, cancer, and celiac disease [3,6].

Compared to other cereals, oat contains a unique group of phenolic alkaloids known as avenanthramides (AVNs). They were originally identified as phytoalexins produced by the plant in response to exposure to pathogens, such as fungi [3]. Structurally, AVNs contain an anthranilic acid moiety conjugated to a phenylalkenoic acid moiety through an amide bond. Two nomenclatures for describing different AVN congeners have been developed: Collins’ and modified Dimberg’s. While the Collins nomenclature denotes AVNs by a combination of numbers (anthranilic acid moiety) and letters (phenylalkenoic acid moiety), Dimberg’s notation consists only of letters [7]. According to the latest research, there are at least 35–40 AVN congeners present in the oat grain and among them, AVNs 2p, 2f, and 2c are the most abundant in oat grains [8]. These AVNs are constitutively expressed in the kernel, reaching the highest concentration in bran, and appear in almost all milling fractions. A recent study [7] found specific distribution patterns of AVNs in oat grain which varied with cultivar and individual AVNs. An in vitro study [9] illustrated that AVNs provide antioxidant activity. AVN 2c displayed the strongest antioxidant activity, followed by AVN 2f and AVN 2p [10]. Moreover, AVNs modulate multiple biological events, resulting in anti-inflammatory anti-itching and immunomodulatory effects. These compounds also exert antiproliferative effects, which help to prevent or treat cancer [11]. The use of high-resolution LC-MS technology currently allows the monitoring of a wider range of AVNs compared to conventional liquid chromatography, which is less sensitive and poorly separates minor AVN types from the matrix [12].

A number of studies confirmed a high range of AVN concentration in oat cultivars ranging from tens to several hundreds of milligrams per kilogram [13,14]. On the other hand, only a limited number of detailed information is available about the impact of location and weather conditions on AVN grain concentrations. Peterson et al. [15] observed that oats grown in a location with warmer temperatures had a higher AVN content. A study by Li et al. [14] observed that a combination of moderate rainfall, accumulated temperature, and radiation enhanced the concentrations of total AVNs. Multari et al. [4] summarized in this context that location, climate, variety, processing methods, and their interactions are all factors playing a significant role in the process of AVN biosynthesis. On the other hand, Rao et al. [16] reported in a recent study that oats grown in areas with higher precipitation and lower temperatures showed an increase in phenolic compounds, including AVN. The comprehensive genetic and environmental study further confirmed that all three major AVNs were heritable, and the estimated heritability was in the range 0.34, 0.39, and 0.41 for AVN 2c, AVN 2p, and AVN 2f, respectively [17]. Simultaneously, the 11-fold range in AVN concentrations in the 100 genotypes studied provided evidence that variability for AVN concentration should allow breeding progress for a higher AVN concentration. Despite the above studies, breeding practices do not yet have prospective donors or a suitable selection and cultivation strategy to intensify the content of AVNs in grains. Simultaneously, the possible influence of the growing system on the AVN content was presented to a limited extent as well. To date, only one study [18] did not confirm the effect of the growing system and the content of the three most abundant AVNs.

The aim of our presented work was to explain the variability of a wider spectrum of eight AVN oat grains in the three-year period against the background of a different cultivar, growing system, and specific central European weather conditions. The main effort was dedicated to calculating predictive regression models in order to look for the dependence between weather conditions (temperatures and precipitation) and variability Σ AVN, which can be considered, according to our findings, as completely original and unpublished information. At the same time, the work further focused on the evaluation of the mutual relations of AVNs to selected important technological and nutritional parameters of oats in order to find possible perspective correlations usable in current oat breeding programs.

## 2. Results

### 2.1. Variability of AVN Contents Compared to Other Oat Grain Parameters

A three-year evaluation of five oat cultivars grown in two different cropping systems and at two different localities in the Czech Republic confirmed a broad concentration range in the detected eight avenanthramides (AVNs). Σ AVNs fluctuated from 25 to 407 mg/kg of dry weight (dw), and the level of relative variability (RSD) of individual AVNs varied from 72 to 114%. This is up to seven times more compared to, e.g., the RSD value found for the content of proteins or β-D-glucan (Table 1). However, these high values also reflected, in some cases, a wider range of intra-variability (RSD_intra_) in four repeated ∑ AVNs assessments (see supplementary Figure S1). The range RSD_intra_ was from 1.4 to 32.6%, with 67% of cases not exceeding RSD_intra_ = 15%.

The three major AVNs (AVN-2p, AVN-2f, and AVN-2c) accounted for about 2/3 of the share of all eight AVNs detected. On average, AVN-2f (47.2 mg/kg of dw) had the highest values in the tested group. In contrast, the lowest mean value was confirmed for AVN-3p (1.0 mg/kg of dw).

Similar relative variability (RSD = 74.8%) as for the Σ AVN was confirmed only in the content of immunoreactive avenin peptides G12. The achieved average value of the tested set (11.2 mg/kg of dw) was significantly below the permitted value of 20 mg/kg for coeliac patients. However, in some individual cases (e.g., ´Patrik´ 2018—locality CB, ´Patrik´ 2019—locality PR, and ´Seldon´ 2019—locality PR), this limit was slightly exceeded (25–40 mg/kg of dw).

The total average values of other monitored parameters, including grain yield and its qualitative parameters (TGW, content of crude proteins, fat, starch, β-D-glucan, and ash), varied to a much lesser extent (from RSD = 4.3% for starch to RSD = 40.3% for yield; see Table 1). In summary, the values for most parameters were typical of oats. On average, higher values were mainly detected in the protein content (CPmax = 20.1% and both protein fractions, AVEmax = 3.5%; GLUmax = 3.5%). A summary of all detected parameters depending on the cultivar, locality, growing system, and year is further presented in Supplementary Tables S1 and S2.

### 2.2. Analysis of the Influence of the Main Factors on the Monitored Grain Parameters

The significance of the four individual factors (cultivar, cropping system, locality, and year) was calculated using a four-way analysis of variance (ANOVA). The variability of all AVNs and most other parameters was significantly affected by all four factors (Table 2 and Table 3). The only exceptions were the three parameters—ash, G12, and β-D-glucan, where the cropping system was not statistically significant, and in the case of β-D-glucan, the locality was also insignificant (Table 3).

**Figure 1:**
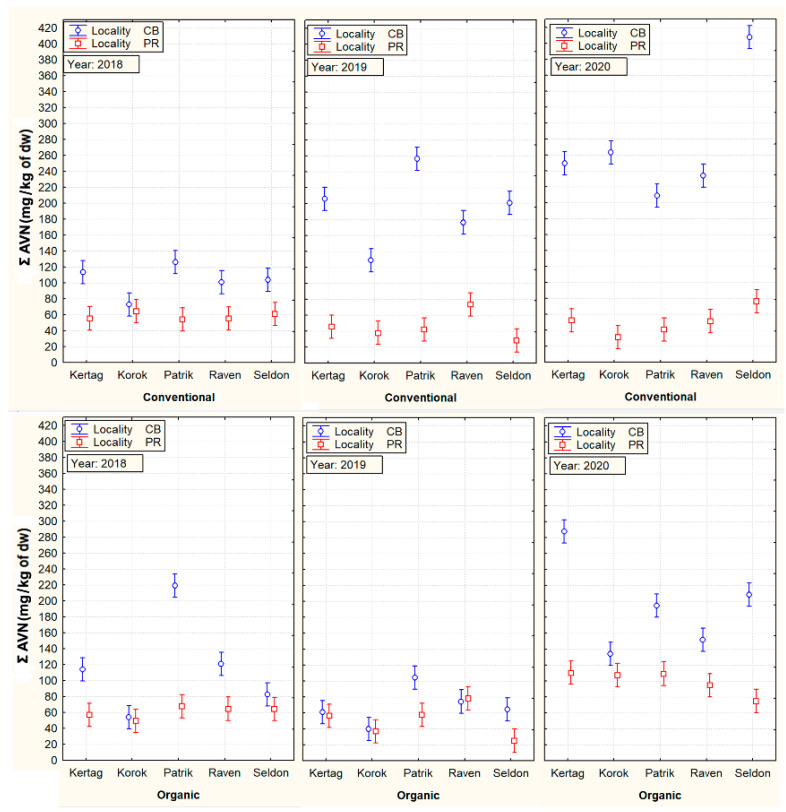
Mutual cultivar, cropping system, locality, and year interactions with total content of tested aventhramides (Σ AVNs). Vertical bars denote 95% confidence intervals.

Statistical analysis of the effects of the main factors on the content of AVNs (ANOVA) and also a subsequent graph describing their mutual interactions are illustrated in Table 2 and Figure 1. The analysis confirmed a higher influence of locality and year on AVN variability compared to cultivar and the cropping system. In particular, the CB locality generally showed higher contents of Σ AVN compared to PR (158.6 mg/kg of dw vs. 60.8 mg/kg of dw). In the conventional conditions of 2019 and 2020, the increase of Σ AVNs compared to the PR locality was even 3–4 times.

**Figure 2:**
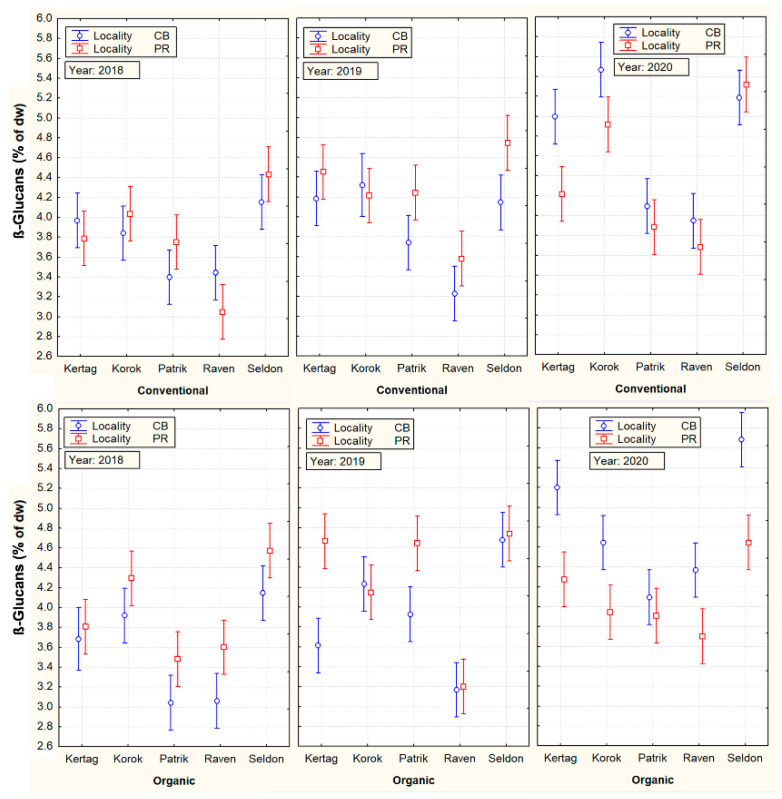
Mutual cultivar, cropping system, locality, and year interactions with ß-D-glucan content. Vertical bars denote 95% confidence intervals.

On the contrary, an example of a grain parameter with a high influence of genotypes (cultivar) on its variability was evident in the content of β-D-glucan, presented in Figure 2.

The three oat cultivars confirmed similar average values of Σ AVNs (116–123 mg/kg of dw), which were significantly higher compared with ´Korok´ (84.9 mg/kg of dw) or ‘Raven’ (106.3 mg/kg of dw) cultivars. Significant differences between individual types of AVNs were also found in the above three cultivars with the highest content of the sum of AVNs (Table 2). The results further showed a significantly higher content of Σ AVNs in the case of conventional cultivation (Table 3). This difference was mainly due to the detected high content of Σ AVN in cultivars at the conventional CB locality in 2019 and 2020. This is especially true for the ‘Seldon’ cultivar, which in 2020 showed an extremely high increase in the content of AVNs (407 mg/kg) in conventional conditions of the CB locality.

**Figure 3:**
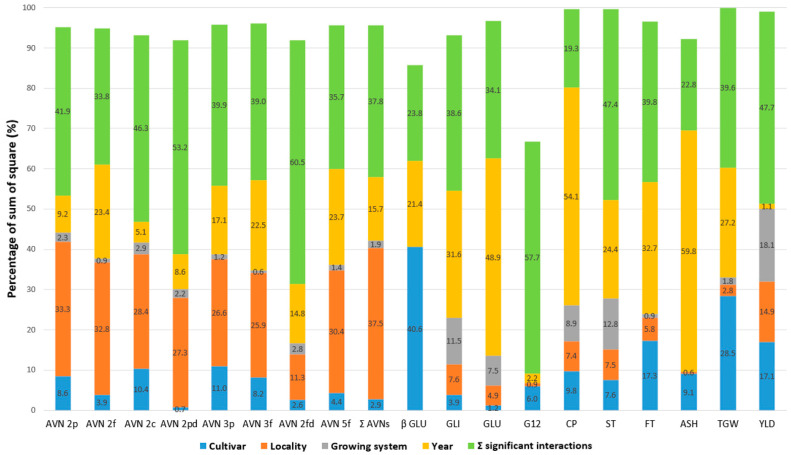
The share of the main significant factors and interactions in the total sum of squares (%) according to analysis of variance (ANOVA) model for selected grain parameters of five oat cultivars grown at two localities and different cropping systems over 3 years. Avenanthramide (AVN), total content of tested AVNs (Σ AVNs), crude protein (CP), starch (ST), fat (FT), ß-D-glucan (ß-GLU), avenin protein fraction (AVE), glutelin protein fraction (GLU), immunoreactive avenin peptides (G12), ash (ASH), thousand-grain weight (TGW), yield (YLD).

A direct quantitative comparison of the percentage influence of significant factors on the variability of the tested parameters (the share of the main significant factors in the total sum of squares) is illustrated in Figure 3. The results confirmed that the contents of individual AVNs were most affected by the locality (26–38%) and then by the year (9–24%). The only exception was AVN-2fd, with a lower total influence of all four main factors (only 31.5%) and the highest influence of the year (14.8%). The influence of the other two factors on the variability of individual AVNs was significantly lower, with ranges of 1–11% for the cultivar and 1–3% for the cropping system, respectively. For Σ AVNs, the effects of the cultivar and cropping system were only 3 and 2%, respectively.

As Table 2 has already indicated, the percentage effects of the main significant factors for the other parameters were different (Figure 3). In the case of β-D-glucan, TGW, and G12, the main dominant factor for their variability was cultivar (40.6%, 28.5%, and 6.0%). The year as the most important factor was found in the CP (54.1%) and ASH (59.8%) parameters. The cropping system was then most significantly reflected in the variability of yield (18.1%), protein content (8.9%), and starch (12.8%). The low percentage effect of all major significant factors (9.1%) and a higher proportion of the sum of significant interactions (57.7%) are evident in the content of immunoreactive avenins (G12). Nevertheless, the effect of the sum of all factors (including their interactions) on the variability of this parameter was significantly lower compared to the others (Figure 3).

### 2.3. Mutual Correlations between AVNs and Other Grain Parameters of Oat

**Figure 4:**
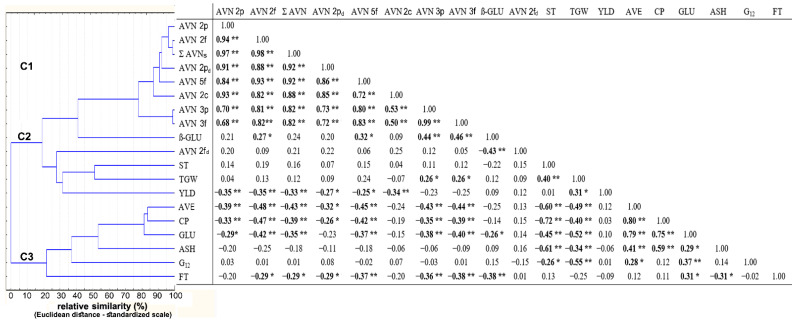
Correlation matrices with hierarchical clustering based on Pearson correlation coefficients, * significant at *p* ≤ 0.05; ** significant at *p* ≤ 0.01. Avenanthramide (AVN), total content of tested AVNs (Σ AVNs), crude protein (CP), starch (ST), fat (FT), ß-D-glucan (ß-GLU), avenin protein fraction (AVE), glutelin protein fraction (GLU), immunoreactive avenin peptides (G12), ash (ASH), thousand-grain weight (TGW), yield (YLD). Statistically significant correlation are in bold.

**Figure 5:**
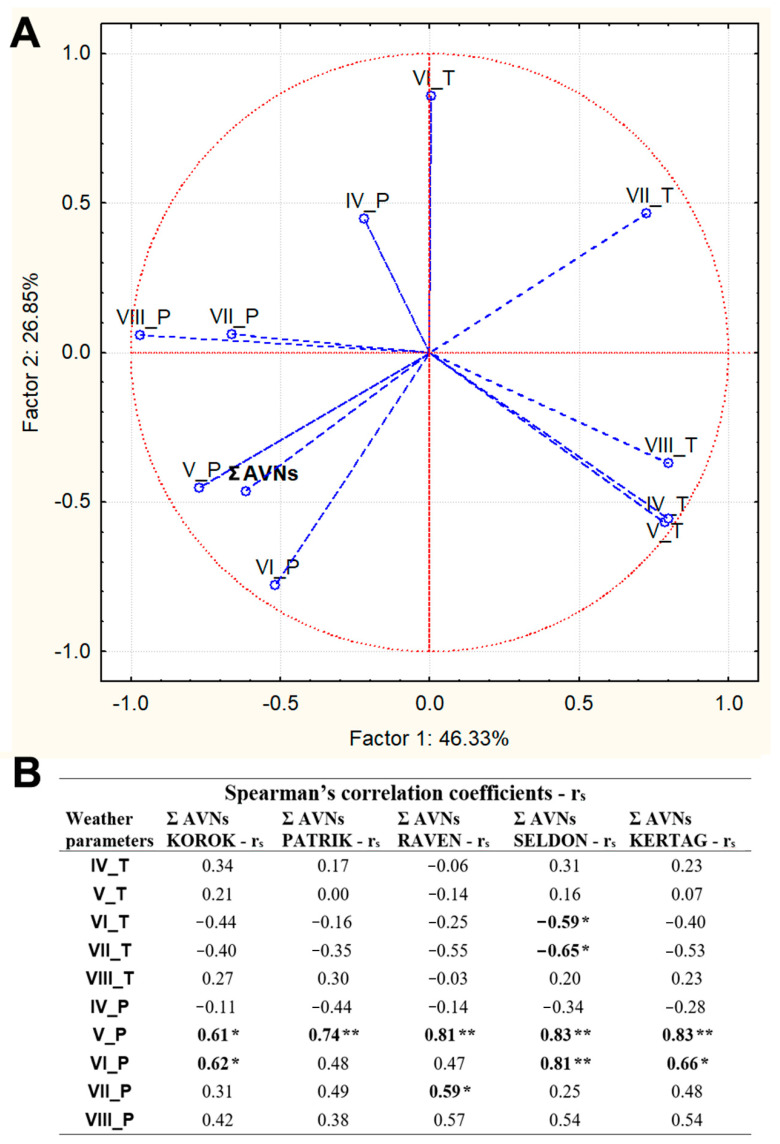
(**A**)The projection of variables (weather parameters vs. Σ AVNs in all cultivars) on a plane of the first and second factor of Principal Component Analysis (PCA). (**B**) Spearman’s correlation coefficients between Σ AVNs in individual oat cultivars and selected weather parameters. Description of symbols: Roman number (month); Sum of precipitation—P; Average temperature—T.; * statistically significant correlations at *p* ≤ 0.05; ** statistically significant at *p* ≤ 0.01. Statistically significant correlations are in bold.

The contents of most AVNs were closely correlated with each other (r = 0.68–0.99; Figure 4). Only AVN-2fd showed a specific low insignificant correlation (r ≤ 0.25). Pearson’s correlations of most AVNs to other monitored grain components were low to moderately strong (0.20 ≤ |r| ≤ 0.48). Significant negative correlations of most AVNs (excluding AVN-2fd) were recorded only for yield, protein content, and protein fractions (−0.48 ≤ |r| ≤ −0.26). Furthermore, the group of four AVNs (AVN 2f, AVN 5f, AVN 3p, and AVN 3f) significantly (positively) correlated with β-D-glucan (0.27 ≤ |r| ≤ 0.43) (Figure 5). Only the mentioned AVN-2fd showed a very specific (negative) correlation to β-D-glucan (r = −0.43). Cluster analysis then graphically classified the tested parameters into three more separate clusters C1–C3 according to mutual correlation coefficients (Figure 5). The cluster C1 grouped Σ AVN with a group of seven individual types of AVNs (except AVN-2fd). The cluster C2 was composed of a set of five following parameters (β-GLU, AVN-2fd, ST, TGW, and YLD). The more separate cluster C3 contained highly correlated parameters of CP and protein fractions avenins (AVE) and glutelins (GLU), to which the parameters ASH, G12, and fat content (FT) were assigned. Despite the high variability of AVNs due to external (non-genetic) factors, their negative correlations to the increasing content of CP and conversely a positive correlation to β-D-glucan (except minor AVN-2fd) is evident.

It is also interesting to compare the low but significant correlations of both storage protein fractions (AVE and GLU) to the content of immunoreactive avenins (G12). Although the reactive (homologous) components of gluten are detected in the alcohol-soluble avenin fraction using the test kit AgraQuant Gluten G12 (see the manufacturer’s protocol), a higher significant correlation to G12 content was confirmed with the content of soluble glutelins (rGLU = 0.37 vs. rAVE = 0.28) (Figure 4).

### 2.4. Effect of Weather Conditions on the Variability of AVNs

Principal component analysis (PCA) and Spearman’s correlations (Figure 5) were used to estimate and illustrate the relationships between Σ AVNs and selected weather parameters on the background of all tested oat cultivars, both cultivation systems, different localities, and three years. Both principal components explained together 68.8% of the total variability (the first: 41.78%, the second: 27.02%).

Principal component analysis (PCA) and Spearman’s correlations (Figure 5A,B) were used to estimate and illustrate the relationships between Σ AVNs and selected weather parameters. In the case of PCA analysis (5A), the mutual relationships are summarized on the background of all tested oat cultivars, both culture systems, different localities, and three years of evaluation. Spearman’s correlation further describes these relations on the background of five individual oat cultivars (Figure 5B).

Both principal components of PCA explained together 73.18% of the total variability (the first: 46.33%, the second: 26.85%). Closer positive relations to the variable Σ AVNs were mainly confirmed by the sum of precipitation in May (V_P) and June (VI_P). In contrast, the average July temperatures (VII_T) showed an antagonistic relationship to the Σ AVN contents.

Subsequent calculations of Spearman’s correlation coefficients (rs) between Σ AVNs and weather parameters performed for individual cultivars (Figure 5B) confirmed positive, strong, and statistically significant correlations between the sum of precipitation in May (V_P) and the growth of Σ AVNs (0.61 ≤ |r_s_| ≤ 0.83). Positive medium to strong correlations, which were even statistically significant in the case of Seldon, Kertag, and Korok cultivars, were also confirmed by the relationships between the sum of precipitation in June (VI_P) and Σ AVNs (0.47 ≤ |r_s_| ≤ 0.81). It is also possible to mention the trend of antagonistic relations between the average temperatures in June and July (VI_T and VII_T) and Σ AVNs. In the case of the Seldon cultivar, these correlations were even statistically significant −0.65- ≤ |r_s_| ≤ −0.59).

## 3. Discussion

More than 40 distinct AVNs in oat seeds have been published so far [8,19]. However, the levels of these various AVNs in the samples are usually very different [8]. Their levels can be in intervals of more than three orders of magnitude. Therefore, we focused only to assess the eight most commonly abundant AVNs in our samples, whose levels were reported to be usually higher than 1 mg/kg dry weight (dw).

The three main detected AVN concentration ranges (AVN-2P-AVN AVN-2f and AVN-2c) were found in oats [4]. Our previous study [8] revealed in 10 selected varieties of oats both a high proportion of three main AVNs (65–70%) and a range of another 33 AVNs, including the structures of 10 novel AVNs. That study also identified nine additional quantitatively significant AVNs (≥1 mg/kg of dw), which represented approximately 25–30% of Σ AVNs [8]. In the context of these results, it can be estimated that the detected number of eight of the most quantitatively significant AVNs in this study represented the majority of all free AVNs of the tested grain. Chromatographic separation of these eight AVNs is shown in Figure S2.

Immunoreactive avenins (G12), which as a single parameter showed a similar level of variability as AVNs, indicated in some cases a slight exceedance of the concentration limit of gluten (20 mg/kg of dw) safe for celiac patients [5]. In these cases, the possible individual contamination of samples with residual gluten from other cereal species during harvesting or post-harvest operations of the samples cannot be completely ruled out. However, some inaccuracies may be related to the unclear suitability of the diagnostic immunochemical kit for monitoring gluten epitopes in oats. On the other hand, the Spanish authors have successfully used this system to identify oat genotypes with different contents of gluten epitopes. At the same time, the results of the genotypes correlated well with the clinical analysis of blood samples in patients with celiac disease [20]. Vice versa, Gilissen et al. [5] disagreed with the interpretation of the immunogenic responses of the G12 monoclonal antibodies (Anbs) in the case of oat. According to these authors, it could be caused by cross-reactivity with some homological sequences in avenins, which do not have to correspond with clinically verified toxic sequences. Currently, only two adenine sequences, which are perhaps recognized by G12 Anbs, are mentioned as resistant to trypsin and chymotrypsin digestion [21], but these most likely occur in every oat cultivar [5].

**Figure 6:**
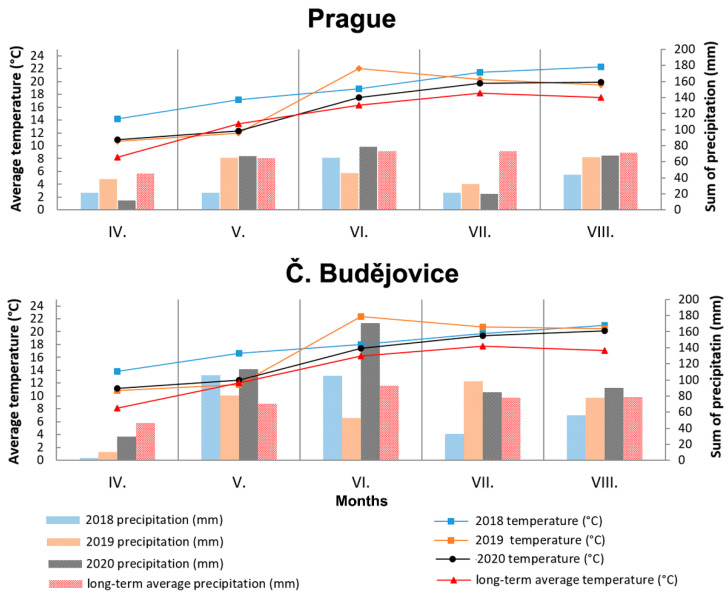
Weather characteristics during vegetation period (April–August) on both localities (Prague and Č. Budějovice) in 2018–2020. Roman numbers (IV.-VIII.) mean months (April–August) of the year.

The total average values of other monitored oat parameters (Table 1), including grain yield and its qualitative parameters (TGW, content of crude proteins, fat, starch, β-D-glucan, and ash) corresponded to published values [22,23]. A 2–3% higher content was on average detected in the avenin fraction–AVE (about 15% in crude protein) compared to the results of Van den Broeck et al. [24]. Concentrations of extractable glutelins (GLU) were similar to those of AVE with a higher RSD value (20.9%). Additionally, in this case, these values were higher compared to the review publication [25] mentioning the proportion of extractable GLUs below 10% in total protein. The main cause of higher contents of the mentioned protein fractions as well as crude protein can probably be found in connection with significantly higher vegetation temperatures in all monitored years in comparison with long-term averages in both localities (Figure 6). As previously mentioned by Capouchová et al. [26], the effect of higher temperatures is manifested by increased plant respiration, which reduces the amount of carbohydrate assimilated and thus increases the percentage of proteins.

The results of ANOVA analysis confirmed the significant effects of all four factors in the case of AVNs. Several authors have reached similar conclusions about the influence of genetic and a number of non-genetic factors on the concentration of AVNs [4,15,16]. In particular, our results corresponded well with a study by Oraby and Ahmed [27] which demonstrated that harvesting years and planting locations affected by higher levels of biotic and/or abiotic stresses induce higher levels of AVNs, nevertheless in a genotype-dependent manner.

One aspect that is very interesting is the mentioned significant increase in the content of AVNs only at the conventional locality in CB in 2019 and 2020. It can be assumed that generally higher plant density in a conventional cropping system, combined with a higher sum of precipitation in July and August 2019 and 2020 (Figure 6), could generate more favorable microclimatic conditions for potential stressors, causing an increase in AVNs. Tybursky et al. [28] also mentioned in this context that the strict adherence to good agricultural practices in organic farming (such as good sowing practices, organic fertilization increasing the diversity of soil micro-organisms) should significantly protect crops from pathogen attacks. Since no other pesticide treatment other than herbicides has been applied in the conventional cropping system, a higher pressure of pathogens in the conventional cropping system is associated with an increased response of AVNs as protective phytoalexins can generally be expected. On the other hand, there was no visible difference in the health status of the plants kept under conventional or organic conditions. At the same time, the trend of AVN content in oat cultivars in conventional and organic cropping systems at the PR locality was rather the opposite (Figure 1).

The above-detected effects of the four main factors on other parameters (Table 3) corresponded to a number of scientific studies. For example, in accordance with other scientific studies [29,30], the contribution of mineral nitrogen in the conventional cropping system had a positive effect on the growth of CP, storage protein fractions (AVE and GLU), TGW, and yield of grain (YLD). On the contrary, the starch content decreased. The significant effect of the cultivar as well as significant annual differences in β-D-glucan content were also confirmed [31]. These authors also mention the significant effect of mineral nitrogen fertilization on the increase of β-D-glucan. However, this was not confirmed by our results.

The evaluation of the percentage influence of significant factors (Figure 3) confirmed the high and—at the same time—specific level of the influence of individual main factors (including their interactions) on the variability of the monitored parameters. The mentioned exception was only the content of immunoreactive avenins (G12), indicating a certain level of undefined variability. This fact can be caused by a lower degree of epitope specificity of the oat avenin kit [5]. Another source of uncertainty in the G12 content is the lower but significant positive correlation of the glutelin fraction with the G12 content (Figure 4). The presence of a variable proportion of the glutelin fraction with reactive groups of peptides in the isolation of avenines using the commercial ELISA test kit AgraQuant cannot be completely ruled out so far. Despite the very low celiac reactivity of oat protein, which is generally safe for celiacs, more attention should be paid to this glutelin fraction.

According to our findings, studies correlating a wider range of AVNs to selected nutritional and technological parameters of oat grain are not yet freely available. Studies explaining the causes of significant variability in the content of AVNs in multi-year field experiments are also very limited. Due to the large influence of non-genetic factors (especially weather conditions), the lower mutual correlations between AVN and other grain parameters (Figure 4) are not surprising. It is also evident that the analysis of a larger number of oat cultivars (genotypes), in combination with greater environmental variability, will be necessary to further refine these correlations. Despite these facts, the significant effect of cultivars on the content of AVNs and the detected positive significant correlation of some AVNs to genotype-highly dependent β-D-glucan [23] could be one of the breeding strategies for obtaining oats with improved nutritional quality.

PCA analysis and Spearman correlations based on individual cultivars confirmed that higher sums of precipitation, especially in May and June, have a significant effect on the increase of Σ AVN in hulled and naked oats (Figure 5A,B). Due to the highly above-average temperatures in all three monitored years, higher precipitation amounts could have an essential effect on plant development and the beginning of grain formation, including the synthesis of AVNs.

Some major differences in the content of Σ AVN between the organic and conventional cropping system at the identical locality and year (e.g., CB in years 2019 and 2020, see Figure 1) also indicated that precipitation probably evoked other effects (stressors), which increase Σ AVNs further accelerated. The scientific literature mentions two main factors is a possible high increase in the content of Σ AVNs. The first factor (stressor), to which more humid conditions suit and which has been shown to increase contents of AVNs, are fungal diseases, e.g., oat rust [32]. However, a higher incidence of fungal diseases in the CB locality in the conventional variant compared to the organic system was not recorded.

The high sum of precipitation in the final stage of grain ripening, often in combination with lodging, can cause the pre-harvest sprouting of grains. Thus, it can be another factor that could significantly increase the Σ AVN content. Significant increases of Σ AVNs in germinated grain have been confirmed from a number of studies [33,34]. Although the individual Spearman correlation coefficients between Σ AVNs and the sum of precipitation in July and August (VII_P and VIII_ P) showed trends of positive moderately strong correlations (Figure 5B), in our experiments, the visual control of grains did not reveal potential latent pre-harvest sprouting. On the other hand, the above-mentioned higher density (interconnection) of plants in the conventional crop system could, due to above-average precipitation, generate a wetter microclimate, evoking latent preharvest sprouting more easily. Preharvest sprouting of grain, which significantly damages starch granules and other important technological parameters of cereals, is not commonly monitored in oats in comparison with wheat and rye [35]. The quantification of damaged starch using the Megazyme starch damage kit was evaluated as part of a nutritional analysis of oats [24]. However, the detected ranges of 1.8–4.0% were not related to the definition of any technological limit or possible changes of bioactive compounds. Despite as yet probably unpublished relationships between the level of damaged starch and the AVN content, a close positive correlation can be expected.

Based on these results, it can be concluded that a basic accelerator of AVN growth in oat grain will be above-average rainfall during grain ripening. Nevertheless, precipitation will probably only generate the basic conditions for the development of other factors (abiotic and biotic stressors), which further trigger the direct synthesis of AVN in oat grain.

## 4. Materials and Methods

### 4.1. Field Experiments

The exact field experiments with 5 selected oat varieties were carried out in the Czech Republic in two different localities in two cropping systems during three vegetation periods in 2018–2020. We obtained 60 combinations of samples (cultivar—5, locality—2, cropping system—2, and year—3) which were measured in 4 replicates. In total, 240 oat samples were evaluated in this study.

The first locality included the experimental plots of the Czech University of Life Sciences in Prague Uhříněves–PR (50°02′00.4″ N; 14°36′32.9″ E) with an altitude of 295 m above sea level, an average annual temperature of 8.4 °C, and a long-term sum of precipitation of 575 mm. The soil classification is clay loam soil.

The next experimental locality was situated in southern Bohemia on experimental plots of the University of South Bohemia in České Budějovice–CB (48°97′47.4″ N; 14°44′75.1″ E) with an altitude of 388 m above sea level, average annual temperature 8.5 °C, and average total precipitation 627 mm. The soil classification is pseudogley, sandy loam.

The oat collection involved four different hulled oat cultivars (*Avena sativa* L.) of the Czech origin ’Korok,’ ‘Kertag,’ ‘Seldon,’ and ‘Raven,’ with the black seed and one naked oat cultivar (*Avena sativa* L.) of the Czech origin ‘Patrik’. Field trials were performed under both organic and conventional growing systems, using red clover (Prague) and legume/cereal mixture (České Budějovice) as preceding crops for oats. The organic crop stands were treated in compliance with European legislation (European Council (EC) Regulation No. 834/2007, the EC Regulation No. 889/2008). No additional fertilizers or pesticides were used in the organically cultivated oat. Nitrogen (60 kg N/ha), along with herbicide, was applied to the oat cultivated conventionally. Randomized blocks in four replicates were designed with an average experimental plot area of 12 m^2^. The crop was harvested at full maturity.

After harvest, the yield of grain reported at 14% moisture and TGW (Thousand Grain Weight) were determined. Then, the oat grains of the hulled varieties were dehulled using a laboratory dehuller Codema LH 5095 (Maple Grove, MN, USA) and prepared for future analyses.

### 4.2. Weather Conditions

Weather vegetation conditions (April–August) at both localities in individual years (2018–2020) are shown in Figure 6. The course of mean monthly temperatures in both localities was similar and all 3 years (2018–2020) in the Czech Republic can be described as very warm compared to the long-term temperature average. However, significant differences between the two localities can be found in the monthly sum of precipitation for vegetation, which was significantly higher at the locality in CB. In particular, the sums of precipitation in May exceeded the sum of precipitation at the PR locality in all years, as well as the long-term average of the locality in the CB. The sum of temperatures during the vegetation period showed that the locality in the PR was warmer by an average of 1 °C only in 2018. In the following years, the sum of vegetation temperatures was comparable between the two localities. However, the achieved ranges of total precipitations 176 mm (2018), 251 mm (2019), and 249 mm (2020) for PR locality lagged significantly behind the CB locality with total precipitations 302 mm (2018), 320 mm (2019), and 488 mm (2020).

### 4.3. Basic Quality Parameters of Oat Grain

Analyses included quantification of following basic quality parameters of the oat grain: content of crude protein (CP) according to Kjeldahl method [36] on Kjeltec KT 200, FOSS, Sweden, and the protein content was calculated by multiplying the nitrogen content by 6.25, content of starch (ST) according to Ewers’ polarimetric method [37] on Polamat A, Carl Zeis Jena, Germany, content of fat (FT) according to Randall method [38] on Randall Hot Extraction apparatus E6, Behr Labor Technik GmbH, Düsseldorf, Germany, and ash content (ASH) after burning at 750 °C for 4 h [39]. The dry matter content of seed samples (5 g) was further dried in an electric hot-air drier at 105 °C for 4 h, according to the standard method [40].

### 4.4. Analyses of Specific Nutritional Parameters of Oat Grain

Analyses of 8 selected most abundant avenathramides defined according to Dimberg’s nomenclature (AVN 2c, AVN 2f, AVN 2p, AVN 2fd, AVN 2pd, AVN 3p, AVN 3f, and AVN 5f) were carried out according to Jágr et al. [8]. Briefly, oat flour was extracted with 80% MeOH solvent, then it was filtered and analyzed using UHPLC system (Dionex, UltiMate 3000 UHPLC system, Dionex Softron GmbH, Germany) coupled with QExactive Orbitrap mass spectrometer (Thermo Fisher Scientific, Rockwood, TN, USA). Chromatographic separation of AVNs was realized using gradient elution with 0.1% formic acid in water as a solvent A and methanol with 0.1% formic acid as a solvent B. LC gradient: 0 min: 75% of solvent A + 25% of solvent B, then linear gradient in 11.0 min: 25% A + 75% B. Column was flushed with 100% of B in 12.0 min and then column was equilibrated back with 75% A and 25% B in 15.0 min. The column was maintained at 40 °C at a flow rate of 0.35 mL/min. MS detection was performed using electrospray ionization in positive mode. Identification of AVN compounds in samples was based on their retention times and on mass spectral data obtained by UHPLC-MS/MS, which were compared with those described in our previous study [8]. The amounts of AVNs in the samples were calculated using calibration curves generated by the analysis of three external standards (AVN 2c, AVN 2f, and AVN 2p) purchased from Sigma-Aldrich (St. Louis, MO, USA). These standards were also used for the semi-quantitative analysis of all the other AVNs (AVN 2fd, AVN 2pd, AVN 3f, AVN 3p, and AVN 5f), for which no commercial authentic standards were available.

Contents of β-D-glucan (β-GLU) were measured by the enzymatic method ‘Mixed-linkage beta-glucan assay procedure’ from Megazyme International Ireland [41] in methodical accordance with Havrlentová et al. [31].

Immunoreactive gluten (avenin) peptides (G12) were assessed using ELISA test kit AgraQuant Gluten G12 (Romer labs Diagnostics) according to the manufacturer’s protocol. All ELISA measurements were in a separate room to avoid gluten contaminations. The absorbance was recorded at 450 nm using Sunrise (Tecan) microplate reader [42].

Both seed storage protein fractions prolamins (avenins—AVE) and glutelins (GLU) were extracted by slightly modified Osborn’s method [43]. Briefly, avenins were extracted with 5 mL 70% (*v*/*v*) aqueous ethanol (50 mg of wholemeal flour; extraction 4 h in 20 °C; permanent mixing; final centrifugation–RCF 3500× *g*). Glutelins were determined by extraction with DTT solution composed of 0.08 M Tris-HCl, (pH 7.5) in 50% 1-propanol with 1% DTT (extraction 1 h in 4 °C; permanent mixing; final centrifugation–RCF 3500× *g*). A 1:10 (*w*/*v*) solids-to-liquid ratio was used for the extractions.

The separation of both protein fractions was determined by high-performance liquid chromatography (RP-HPLC) [44]. Protein extracts (10 μL) were injected into RP-HPLC using a Waters 2965 apparatus with UV detector and 300 SB-C8 Zorbax Poroshell ™ column (75 × 2.1 mm, 5 µm particles) linked to a Zorba 300SB-C8 cartridge guard column (Rockland Technologies, Inc., Newport, DE, USA). Elution of avenins and glutelins was monitored at the wavelength 210 nm. Wheat gliadin (Sigma Aldrich) was used as an external standard for final quantification.

Prolamin (avenin) and glutelin separations were carried out using a flow rate of 1.5 mL/min at 60 °C for avenins [45] and a flow rate of 0.7 mL/min at 60 °C for glutelins [44]. Two mobile phases were used: (A) 0.1% trifluoroacetic acid–TFA (*v*/*v*) in acetonitrile–ACN, (B) 0.1% TFA (*v*/*v*) in deionized water. Both protein fractions were separated by gradient elution adjusted as follows: 0–2.5 min 23% (A) + 77% (B), 2.5–9.5 min 30% (A) + 70% (B), 9.5–13 min 47% (A) + 53% (B), 13–15 min changed to 23% (A) + 77% (B). The linear calibration for quantification of both fractions (AVE and GLU) was in the range of standard wheat gliadin 0.5–5 mg/mL.

### 4.5. Statistical Methods

All chemical and nutritional parameters were assessed in 4 repetitions. Basic statistics included the determination of the mean, standard deviation (SD), standard error (SE), minimum/maximum, and relative standard deviation (RSD) in all 19 tested parameters.

The next statistical methods further included 4-way analyses of variance (ANOVA) followed by Tukey’s Honest Significance Test (Tukey HSD test) to determine significant differences between tested parameter averages. The percentage effect of significant factors was calculated based on the ANOVA as a percentage of the sum of squares (% SS) of the significant factor to the total sum of squares [46].

Pearson’s correlation matrix of individual parameters with mutual hierarchical clustering (standardize Euclidean distance and UPGMA method as a clustering technique) were also used.

The applied calculations of Principal Component Analysis (PCA) [47] and Pearson‘s correlations were used to illustrate the relationship between AVNs as selected parameters of weather conditions. All above mentioned statistical methods were computed with the Statistica 7.1CZ software StatSoft, Inc. (2005) (StatSoft, Tulsa, USA).

## 5. Conclusions

The achieved three-year results confirmed the quantitative differences of a wider spectrum of three main and five minor AVNs between both oat cultivars and individual types of AVNs. Individual AVNs confirmed a very high variability (RDS = 72.7–113.5%), which was dominantly influenced by the locality and year factor. The effect of the cultivar and cropping system was also statistically significant, but with a significantly lower share in the total variability of AVN content. With the exception of AVN-2fd, the other AVN types confirmed high mutual correlations 0.7 ≤ |r| ≤ 0.9. AVNs showed only lower to medium correlations to the other 10 grain parameters. Despite this fact, detected significant correlations (0.27–0.46) between four AVNs (AVN 2f, AVN 5f, AVN 3p, and AVN 3f) and strongly genetically fixed β-D-glucan contents could be effectively used in breeding programs for the synergetic increase of both parameters. PCA analysis and Spearman correlations based on individual cultivars confirmed significant positive relationships between the sum of precipitation in June and July and the increase of Σ AVNs. Precipitation during this period at otherwise very above-average temperatures could favorably affect grain formation as well as AVN synthesis. However, the results also indicate that higher precipitation can also generate favorable conditions for related factors, such as preharvest sprouting, evoking a direct increase in the AVN content of the oat grain.

## Figures and Tables

**Table 1:** Achieved ranges of tested grain parameters and their relative variability (RSD) in selected oat cultivars in the years 2018–2020.

Parameters	N	Mean	Minimum	Maximum	SD	SE	RSD (%)
AVN 2p (mg/kg of dw)	240	20.6	4.3	86.3	15.0	1.9	72.7
AVN 2f (mg/kg of dw)	240	47.2	7.8	174.9	36.4	4.7	77.1
AVN 2c (mg/kg of dw)	240	16.4	3.5	72.6	12.3	1.6	75.2
AVN 2p_d_ (mg/kg of dw)	240	3.7	0.8	19.2	3.0	0.4	80.1
AVN 3p (mg/kg of dw)	240	1.0	0.1	5.3	1.2	0.2	113.5
AVN 3f (mg/kg of dw)	240	10.7	0.7	55.8	11.8	1.5	110.6
AVN 2f_d_ (mg/kg of dw)	240	6.2	1.1	26.6	5.5	0.7	88.6
AVN 5f (mg/kg of dw)	240	3.9	0.5	17.9	3.5	0.5	89.9
Σ AVNs	240	109.7	25.2	407.4	78.8	10.2	71.9
CP (%)	240	16.4	12.5	20.1	2.0	0.3	11.9
ST (%)	240	61.7	54.8	67.0	2.8	0.4	4.5
FT (%)	240	4.8	4.1	7.4	0.6	0.1	13.5
ß-GLU (%)	240	4.1	3.0	5.7	0.6	0.1	14.7
AVE (%)	240	2.6	1.5	3.5	0.5	0.1	17.5
GLU (%)	240	2.5	1.5	3.5	0.5	0.1	20.9
G_12_ (mg/kg)	240	11.2	2.4	39.9	8.4	1.1	74.8
ASH (%)	240	2.3	2.0	2.8	0.2	0.0	7.9
TGW (g) *	240	31.1	20.8	38.6	4.5	0.6	14.3
YLD (t/ha)	240	4.7	1.6	9.4	1.9	0.2	40.3

* Calculated in hulls except for cv. ’Patrik´, Avenanthramide (AVN), total content of tested AVNs (Σ AVNs), crude protein (CP), starch (ST), fat (FT), ß-D-glucan (ß-GLU), avenin protein fraction (AVE), glutelin protein fraction (GLU), immunoreactive avenin peptides (G12), ash (ASH), thousand-grain weight (TGW), yield (YLD), level of relative variability (RSD), standard deviation (SD, standard error (SE), total number of tested samples (N).

**Table 2:** Effects of four main factors and their statistical significance on selected contents of 8 avenanthramides (mg/kg of dw)–AVNs (four-way ANOVA).

Factors		AVN 2p	AVN 2f	AVN 2c	AVN 2p_d_	AVN 3p	AVN 3f	AVN 2f_d_	AVN 5f	Σ AVNs
Cultivar	*F_crit_*	*134.8* **	*56.4* **	*115.2* **	*6.8* **	*14.1* **	*161.1* **	*24.4* **	*75.9* **	*49.8* **
	Kertag	20.2 ^c^	51.3 ^a,b^	12.6 ^a^	3.7 ^a^	1.7 ^d^	16.0 ^d^	7.3 ^b^	4.5 ^a^	117.3 ^a^
	Korok	14.0 ^a^	33.3 ^c^	11.1 ^a^	3.5 ^a^	1.3 ^c^	13.3 ^c^	5.2 ^a^	3.3 ^c^	84.9 ^c^
	Patrik	26.3 ^b^	54.5 ^b^	21.6 ^b^	3.8 ^a,b^	0.8 ^a^	7.8 ^a^	5.9 ^a^	2.8 ^b^	123.3 ^a^
	Raven	17.8 ^d^	48.7 ^a^	16.6 ^c^	3.5 ^a^	0.6 ^b^	7.3 ^a^	7.1 ^b^	4.7 ^a^	106.3 ^b^
	Seldon	24.6 ^e^	48.2 ^a^	20.0 ^b^	4.2 ^b^	0.9 ^a^	9.0 ^b^	5.2 ^a^	4.4 ^a^	116.4 ^a^
Locality	*F_crit_*	*2095.5* **	*1914.4* **	*1255.3* **	*1016.9* **	*1862.7* **	*2025.0* **	*419.4* **	*2093.8*	*2607.7* **
	CB	29.4 ^b^	68.4 ^b^	23.1 ^b^	5.3 ^b^	1.7 ^b^	16.7 ^b^	8.1 ^b^	5.9 ^b^	158.6 ^b^
	PR	11.8 ^a^	25.9 ^a^	9.6 ^a^	2.1 ^a^	0.4 ^a^	4.6 ^a^	4.3 ^a^	2.0 ^a^	60.8 ^a^
Cropping	*F_crit_*	*145.9* **	*53.3* **	*129.0* **	*81.1* **	*83.3* **	*46.4* **	*102.8* **	*97.0* *	*129.2* **
	CONV	22.9 ^b^	50.7 ^b^	18.5 ^b^	4.2 ^b^	1.2 ^b^	11.6 ^b^	7.1 ^b^	4.4 ^b^	120.6 ^b^
	ORG	18.3 ^a^	43.6 ^a^	14.2 ^a^	3.3 ^a^	0.9 ^a^	9.7 ^a^	5.2 ^a^	3.5 ^a^	98.8 ^a^
Year	*F_crit_*	*291.2* **	*681.9* **	*113.7* **	*160.1* **	*597.7* **	*877.7* **	*274.6* *	*814.5* *	*546.6* *
	2018	17.4 ^a^	35.1 ^a^	12.8 ^a^	3.1 ^a^	0.6 ^a^	6.0 ^a^	7.2 ^b^	2.8 ^a^	85.0 ^a^
	2019	17.3 ^a^	33.9 ^a^	16.5 ^b^	3.1 ^a^	0.8 ^b^	7.3 ^b^	8.2 ^c^	2.6 ^a^	89.6 ^a^
	2020	27.1 ^b^	72.5 ^b^	19.8 ^c^	5.0 ^b^	1.7 ^c^	18.6 ^c^	3.2 ^a^	6.4 ^b^	154.4 ^b^

* significant at *p* ≤ 0.05; ** significant at *p* ≤ 0.01; values with different letter indexes are significantly different at *p* ≤ 0.05 (Tukey HSD test). Total content of tested AVNs (Σ AVNs), locality České Budějovice (CB), locality Prague Uhříněves (PR), conventional (CONV), organic (ORG).

**Table 3:** Effects of four main factors and their statistical significance on selected contents of grain parameters (four-way ANOVA).

Factors		ß-GLU (%)	AVE (%)	GLU (%)	G_12_ (mg/kg of dw)	CP (%)	ST (%)	FT (%)	ASH (%)	TGW (%)	YLD (t/ha)
Cultivar	*F_crit_*	*131.8* **	25.7 **	16.2 **	9.7 **	*1088.7* **	*910.9* **	*224.3* **	*53.9* **	*12,214.9* **	*783.1* **
	Kertag	4.2 ^a^	2.5 ^b^	2.4 ^b^	9.8 ^a,b^	15.8 ^a^	62.7 ^e^	4.8 ^a^	2.3 ^a^	32.2 ^a^	5.3 ^c^
	Korok	4.3 ^a^	2.7 ^a^	2.5 ^b^	10.3 ^a,b^	17.5 ^e^	60.4 ^a^	4.5 ^b^	2.4 ^b^	32.9 ^d^	4.8 ^a^
	Patrik	3.9 ^c^	2.6 ^a^	2.6 ^a^	15.1 ^c^	16.0 ^b^	61.3 ^b^	5.3 ^d^	2.2 ^a^	26.4 ^b^	3.3 ^b^
	Raven	3.5 ^b^	2.7 ^a^	2.6 ^a^	8.3 ^a^	16.5 ^d^	62.1 ^d^	4.8 ^a^	2.2 ^a^	31.8 ^c^	4.8 ^a^
	Seldon	4.7 ^d^	2.5 ^b^	2.6 ^a^	12.7 ^b,c^	16.2 ^c^	61.9 ^c^	4.7 ^c^	2.2 ^a^	32.2 ^a^	5.5 ^d^
Locality	*F_crit_*	*0.1*	302.8 **	399.1 **	5.6 *	*3953.0* **	*3615.1* **	*300.3* **	*13.6* *	*4783.8* **	*2742.2* **
	CB	4.1 ^a^	2.8 ^b^	2.7 ^b^	10.4 ^a^	15.9 ^a^	62.5 ^b^	4.6 ^a^	2.3 ^b^	31.8 ^b^	4.0 ^a^
	PR	4.1 ^a^	2.4 ^a^	2.4 ^a^	12.1 ^b^	16.9 ^b^	60.9 ^a^	4.9 ^b^	2.2 ^a^	30.4 ^a^	5.5 ^b^
Cropping	*F_crit_*	*1.5*	201.1 **	263.9 **	0.9	*3284.6* **	*6167.9* **	*47.0* **	*3.5*	*3126.1* **	*3330.8* **
	CONV	4.1 ^a^	2.7 ^b^	2.7 ^b^	11.6 ^a^	17.0 ^b^	60.7 ^a^	4.7 ^a^	2.3 ^a^	31.7 ^b^	5.5 ^b^
	ORG	4.1 ^a^	2.5 ^a^	2.4 ^a^	10.9 ^a^	15.8 ^a^	62.7 ^b^	4.9 ^b^	2.2 ^a^	30.5 ^a^	3.9 ^a^
Year	*F_crit_*	*139.2* **	416.8 **	1308.6 **	7.3 **	*11,990.3* **	*5866.5* **	*849.0* **	*709.3* **	*23,355.1* **	*105.5* **
	2018	3.8 ^a^	2.7 ^b^	2.8 ^a^	10.2 ^a^	16.8 ^b^	63.0 ^c^	5.3 ^c^	2.1 ^a^	32.0 ^b^	4.8 ^a^
	2019	4.1 ^b^	2.9 ^c^	2.8 ^a^	13.3 ^b^	17.9 ^c^	59.8 ^a^	4.6 ^b^	2.5 ^b^	27.9 ^a^	4.4 ^b^
	2020	4.5 ^c^	2.2 ^a^	2.0 ^b^	10.2 ^a^	14.5 ^a^	62.2 ^b^	4.4 ^a^	2.2 ^a^	33.4 ^c^	4.9 ^a^

* significant at *p* ≤ 0.05; ** significant at *p* ≤ 0.01; values with different letter indexes are significantly different at *p* ≤ 0.05 (Tukey HSD test). Crude protein (CP), starch (ST), fat (FT), ß-D-glucan (ß-GLU), avenin protein fraction (AVE), glutelin protein fraction (GLU), immunoreactive avenin peptides (G12), ash (ASH), thousand-grain weight (TGW), yield (YLD), locality České Budějovice (CB), locality Prague Uhříněves (PR), conventional (CONV), organic (ORG).

## Data Availability

The Data from experiments and analyses presented in this study are in a publicly accessible repository available in Supplementary Table S1, Table S2, and Figure S1.

## Supplementary Materials

The following are available online at https://www.mdpi.com/2223-7747/10/11/2485/s1, Table S1: Average contents of eight individual avenanthramides (AVN), including their total concentrations (Σ AVNs) in oat grain depending on the factors of cultivar, year, locality, and cropping system. Table S2: Average contents of the 10 next grain parameters of oats depending on the factors of cultivar, year, locality, and cropping system. Figure S1: Frequency of relative standard deviation (RSD_intra_) of repeated assessments of Σ AVNs (cultivar x locality x farming system x year). Figure S2: Analysis of eight aventhramides (AVNs) in oat seeds by UHPLC/HRMS/MS with MS/MS spectrometer operating in PRM (parallel reaction monitoring) mode.
