# Supplementary figures and images for: Avenanthramides: Unique Bioactive Substances of Oat Grain in the Context of Cultivar, Cropping System, Weather Conditions and Other Grain Parameters
Source: Plants (Basel). 2021 Nov 17;10(11):2485. doi: 10.3390/plants10112485 (PMC8624809; doi:10.3390/plants10112485)

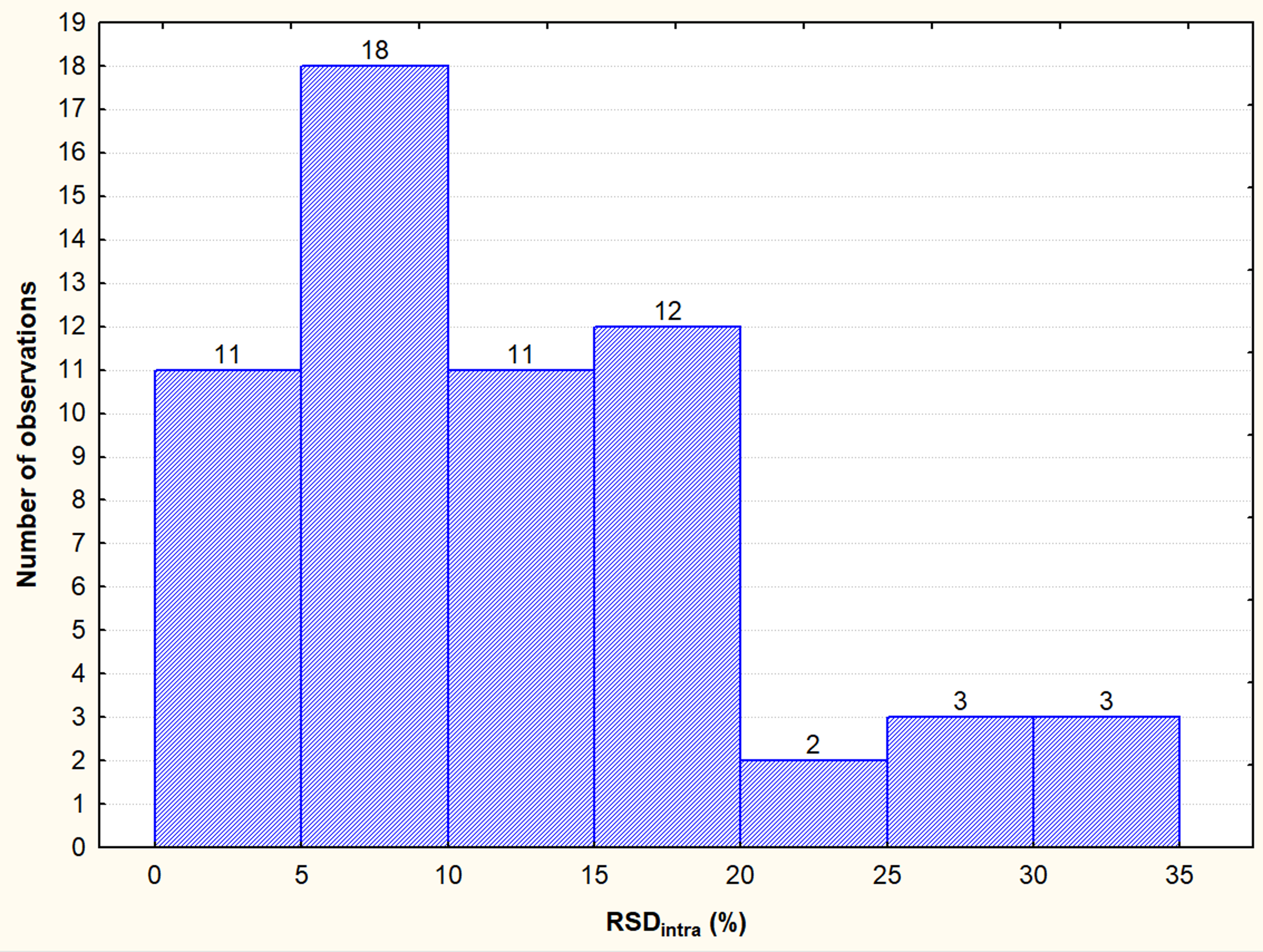

Supplement: Supplementary file 1 [file plants-10-02485-s001.zip › plants-1409821-supplementary/Figure S1.tif]

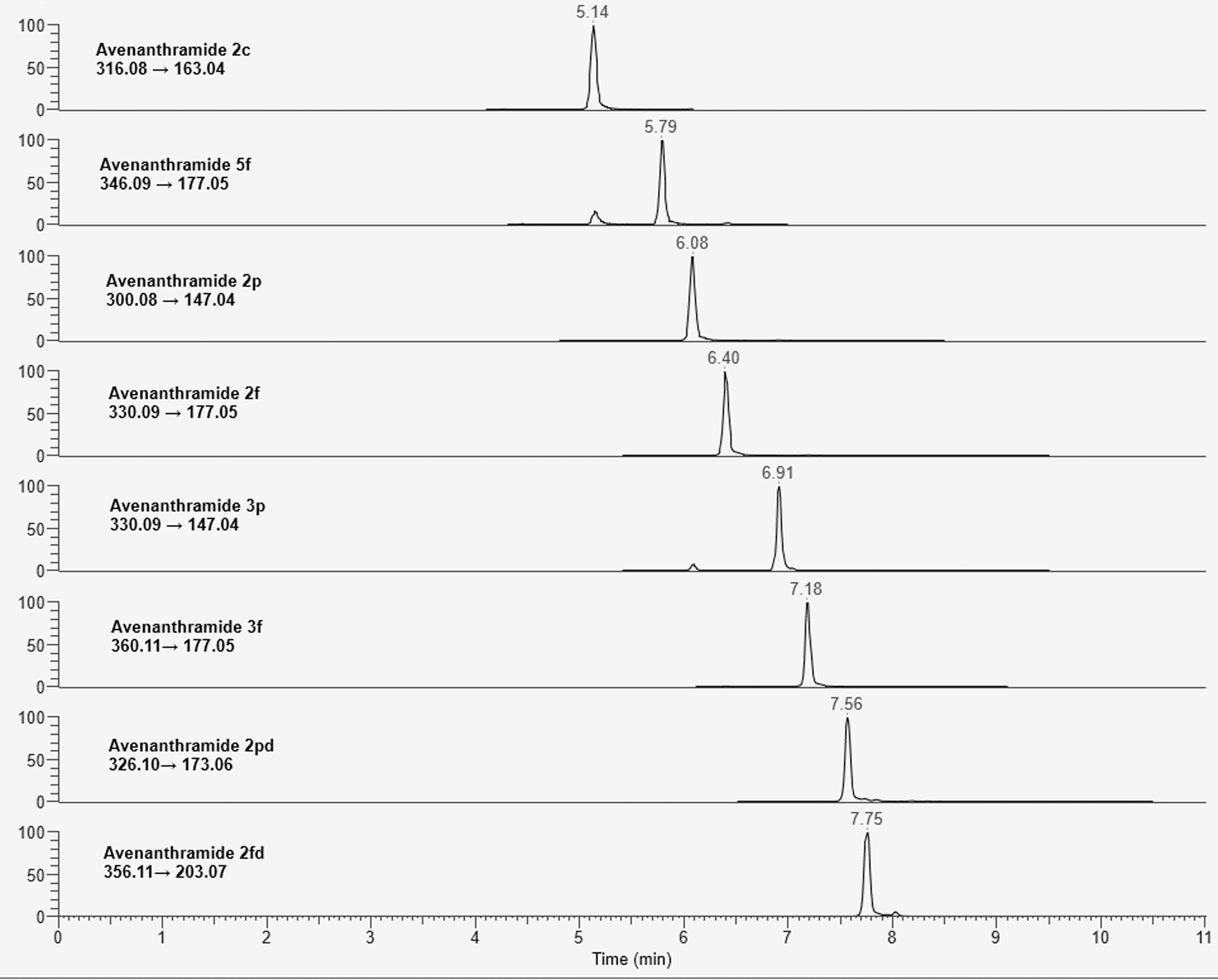

Supplement: Supplementary file 1 [file plants-10-02485-s001.zip › plants-1409821-supplementary/Figure S2.tif]
